# Activity Enhancement of Ferrierite in Dimethyl Ether Carbonylation Reactions through Recrystallization with Sodium Oleate

**DOI:** 10.3390/molecules28135279

**Published:** 2023-07-07

**Authors:** Jiangang Lv, Long Chen, Chong Chen, Yunzheng Wang, Di Wang, Huaqian Sun, Weimin Yang

**Affiliations:** 1 Sinopec Shanghai Research Institute of Petrochemical Technology Co., Ltd., Shanghai 201208, China; lvjg.sshy@sinopec.com (J.L.); chenlong0710@yeah.net (L.C.); chenc.sshy@sinopec.com (C.C.); wangyz.sshy@sinopec.com (Y.W.); wangd.sshy@sinopec.com (D.W.); sunhj.sshy@sinopec.com (H.S.); 2 School of Chemical Engineering, East China University of Science and Technology, Shanghai 200237, China

**Keywords:** ferrierite, dimethyl ether carbonylation, recrystallization

## Abstract

Methyl acetate (MA) has a wide range of applications as an important industrial chemical. Traditional MOR zeolite for carbonylation of DME to MA accumulated carbon easily because of a 12-membered ring (12 MR) channel. In this work, we innovatively developed the method of recrystallization ferrierite (FER) zeolite using special chelating ligand sodium oleate which can affect ions other than alkali metals. The characterization results of N_2_ adsorption, transmission electron microscopy (TEM), and Fourier transform infrared spectroscopy (FT-IR) show that hydrothermal recrystallization of ferrierite using sodium oleate resulted in a higher Si/Al ratio, a bigger specific surface area and a larger number of Brønsted acid sites in the eight MR channels, which was more efficient in the reaction of carbonylation of dimethyl ether than ordinary alkali treatment.

## 1. Introduction

Methyl acetate (MA) has a wide range of applications as an important industrial chemical used in paints, plastics and solvents in many reactions [1,2,3]. Notably, the MA can be transformed to ethanol and methanol by hydrogenation, where methanol can be efficiently recycled by a dehydration reaction. Therefore, the production of highly purified fuel ethanol is realized in this route [4,5,6,7,8,9], and the synthesis method of MA has gained intensive attention. The carbonylation of syngas-derived dimethyl ether (DME) with CO is an environmentally-friendly route for the production of MA, and it is of great importance to the production of fuel ethanol [10]. Noble metal–iodine catalysts (Rh-I and Ir-I homogeneous catalysts) were reported in the 20th century in the industrial application of methanol carbonylation to acetic acid and DME carbonylation to MA [11,12]. However, the high corrosivity of I- and time-consuming separation procedure had limited the development of the process. In 2006, Iglesia and coworkers reported that the acidic zeolites, including MOR with twelve- and eight-membered rings and FER with ten- and eight-membered rings, are active catalysts for the MA formation through the carbonylation of DME [13]. Compared with a traditional organometallic homogeneous catalyst system, zeolite catalysts have enormous advantages in the nonuse of corrosive halide promoters, the realization of continuous plug-flow processing and easy product separation [14,15,16]. MOR zeolite has been widely reported to be the most active and selective catalyst for carbonylation of DME, while its 12-membered ring (12 MR) channel would be easily blocked by coke, which would lead to a dramatic decrease in activity with a prolonged reaction time [6,17,18,19].

Interestingly, the ferrierite (FER) zeolites show good catalytic stability in the reaction, attributing to its smaller pore sizes with 0.54 × 0.42 nm along with [001] direction for the 10 MR channels and 0.4 × 0.35 nm along with [010] direction of the eight MR channels, which can suppress the formation of carbonaceous deposit to some extent [6,20,21]. However, it shows a lower carbonylation activity compared with MOR zeolites, which could be related to the presence of a smaller-sized FER cage [82626458], into which it is difficult for reactants to enter and reach active sites [20]. Therefore, lots of studies devoted to exploring the reaction mechanism of MA carbonylation over MOR and FER catalysts and measuring the quantity of acidic sites in MOR and FER by combining with varied infrared spectroscopy validate that the rate of MA formation is linearly proportional to the number of Brønsted acid sites in eight MR [22,23,24].

To improve the catalytic activity of FER zeolite in the carbonylation of DME, much effort has been devoted to the development of promising FER based catalysts. Using quantum-chemical techniques, Corma’s group found that the unique selectivity for the carbonylation of methanol and dimethyl ether is connected to the pore shape as well as the size of the eight MR channel (the first reaction step happens preferentially within eight MR channels) [25]. Li’s investigation results supported the theoretical analysis results that the eight MR channel is selective for the production and stability of the acetyl intermediate, which was the rate-limiting phase of the carbonylation reaction by characterizing with in-situ 13C solid-state NMR spectroscopy [26]. According to above reports, the catalyst activity was dependent on the amount of Brønsted acid sites in the eight MRs [27,28,29]. Brønsted acid sites in zeolites are commonly generated by the protons balancing the negatively charged framework induced, and thus the key point of enhancing catalytic activity of FER zeolite is to introduce more B acidic sites to the eight MR. Davis and coworkers used a combination of organic structure-directing agents influencing the aluminum distribution which results in the selective population of acid sites in the eight MR channel to change DME carbonylation rates [30]. Bae and coworkers employed the seed-derived FER and recrystallization repetition, discovering it was an effective synthetic strategy to enhance the catalytic activity and stability ascribed to its enhanced crystallinity with the controlled Al atom locations in the eight MR channels of the FERs [28]. The study conducted by Xu et al. demonstrated that mild alkaline treatment yielded higher carbonylation activity in FER compared to the untreated sample. This finding implies that the disintegration of FER clusters and the elimination of amorphous debris from the surface significantly contribute to the exposure of active sites, resulting in enhanced catalytic activity. The removal of these undesired components through alkaline treatment is likely to enhance the accessibility and availability of active sites, consequently improving the performance of FER in carbonylation reactions [1].

In this work, we innovatively developed the method of recrystallization using special chelating ligand such as sodium oleate which can affect ions other than alkali metals [31]; the characterization and activity test results show that the chelating ligand may influence the distribution of Al species in the zeolite channel during the recrystallization procedure, and specifically promote more Brønsted sites to form in eight MR. By chelating the Al species in the hydrothermal system and protecting it from alkali degradation, sodium oleate might change the ferrierite’s acid characteristics during recrystallization. In this way, ferrierites were synthesized which could increase available Brønsted acid sites in the eight MR channels, thus improving the reactivity and stability of the DME carbonylation reaction. Furthermore, this novel approach to the post-treatment of zeolites provides a promising avenue for producing methyl acetate (MA), which is crucial for fuel ethanol production. Overall, our work not only presents a new perspective on zeolite post-treatment but also demonstrates its potential in manufacturing MA for fuel ethanol production.

Moreover, this method provides a new idea of post-treatment zeolite, offering a promising way to manufacture MA for the production of fuel ethanol.

## 2. Results and Discussion

### 2.1. Structure and Texture of Ferrierites

**Figure 1:**
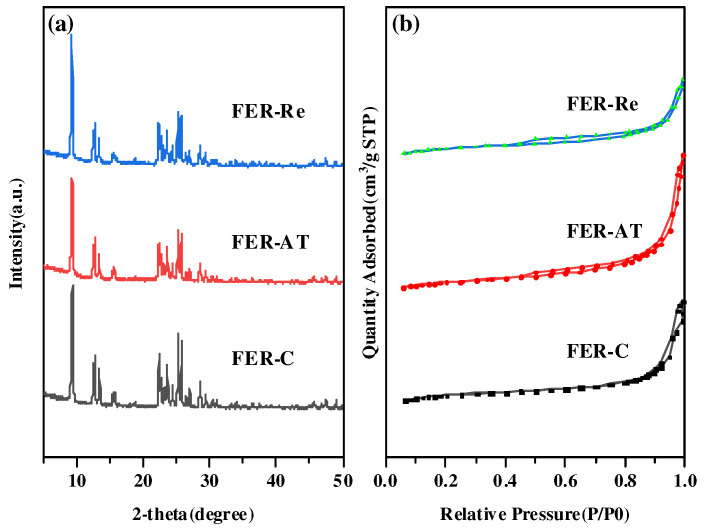
(**a**) XRD patterns of ferrierite catalysts; (**b**) isothermal adsorption–desorption lines of ferrierite catalysts.

The powder XRD patterns of prepared ferrierite catalysts are illustrated in Figure 1a. All samples were highly crystalline pure phase FER zeolites and presented typical diffraction peaks assigned to FER type at 9.2, 25.2 and 25.7. The intensity of the diffraction peaks of FER-AT decreased upon the NaOH solution treatment, indicating a damaged zeolite framework possibly caused by partial dissolution in the alkaline medium. Combined with the ICP results, the Si/Al ratio dropped from 20.7 for FER-C to 18.9 for FER-AT, indicating it had a typical desilication effect of alkaline treatment [23]. After the recrystallization process by adding sodium oleate, the crystallinity of the FER-Re sample increased compared to that of FER-AT (Table 1), showing the recovery of the degree of FER crystallinity to some extent. Moreover, the Si/Al ratio of FER-Re increased to 20.3, which indicates that the removed silicate species may be involved in the reconstruction of the FER framework.

The N_2_ adsorption–desorption isotherms of samples are depicted in Figure 1b. The hysteresis loops are obvious at P/P_0_ ~0.45 of obtained FER-AT and FER-Re due to the small thickness of platelet crystals. These results indicate the mesopores are formed in the ferrierites treated by alkali or sodium oleate. As expected, the external surface area of FER-AT and FER-Re significantly increased, whereas the corresponding micropore area slightly decreases. The textural properties of FER-C, FER-AT, and FER-Re were characterized by nitrogen adsorption–desorption isotherms and the results are listed in Table 1. Compared with FER-C, the microporous surface area and microporous volume of FER-AT are significantly decreased from 342.35 m^2^/g to 289.24 m^2^/g and 0.130 cm^3^/g to 0.104 cm^3^/g, respectively. This is consistent with the above XRD patterns, confirming the destructive effect of the alkali treatment. On the other hand, the external surface area and mesopore volume in FER-AT were increased from 41.91 m^2^/g to 59.10 m^2^/g and from 0.136 cm^3^/g to 0.178 cm^3^/g, respectively, which could be attributed to the formation of mesopores as a result of desilication. After recrystallization, the microporous surface area and microporous volume of FER-Re increased from 230.14 m^2^/g to 279.08 m^2^/g and 0.104 cm^3^/g to 0.124 cm^3^/g, respectively, suggesting that the zeolite structure was repaired. Although the external surface area and the mesopore volume of FER-Re were reduced to 51.01 m^2^/g and 0.160 cm^3^/g, respectively, these values were still higher than those of FER-C, which may be assigned to the filling effect of sodium oleate as a surfactant.

**Figure 2:**
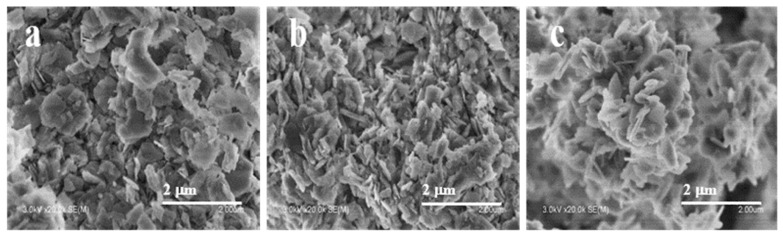
SEM of the ferrierite catalysts (**a**) FER-C; (**b**) FER-At; (**c**)FER-Re.

Scanning and transmission electron microscopes were applied to study the morphological changes shown in Figure 2. As shown, three samples all took the shape of plate-like particles, which are typical morphologies of the FER zeolite [32]. In strong contrast to the FER-C, alkali washing led to an impressive size decrease of the aggregated particles in FER-AT, and amorphous material formed on the external surface of FER-AT that could account for its reduced crystallinity (Figure 2b). As shown in Figure 2c, after recrystallization, the external surface of FER-Re appears smooth and the morphology of FER-Re is regular and uniform, presenting its better crystallinity compared to FER-AT and further proving the zeolite framework has been rebuilt.

**Figure 3:**
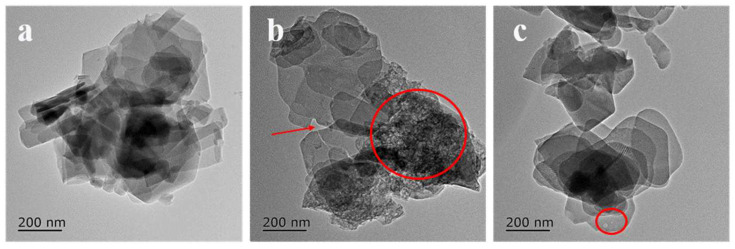
TEM images of the ferrierite catalysts (**a**) FER-C; (**b**) FER-At; (**c**) FER-Re.

More detailed information concerning the structure was obtained by transmission electron microscopy characterization. As can be seen from Figure 3a, some tiny debris exist on the surface of the ferrierite’s platelets on FER-C. These species disappear after alkali treatment, and some amorphous species emerge on FER-AT (Figure 3b), providing direct evidence for effect of alkaline cleaning. Meanwhile, some fissures could be clearly observed which are labeled using arrows in Figure 3b, possibly formed from the attacking of alkali. Moreover, there are a few tiny holes on the surface of FER-At. The pronounced effect of alkaline treatment on FER is the disaggregation of the particles and the creation of some small holes. As presented in Figure 3c, the FER-Re possessed a smooth surface and uniform shape after recrystallization, and the amorphous detritus were removed compared to FER-AT (Figure 3b). Based on the Si/Al ratio in Table 1, the main composition of the alkaline washing debris is Si species on FER-AT. By comparison, this was probably just a process of recrystallizing and smoothing of the surface, and the holes did not get bigger on the FER-Re, most likely because sodium oleate inhibited the attacking of the alkali effect during recrystallization, shown in Figure 3c.

### 2.2. Acid Properties of Ferrierites

**Figure 4:**
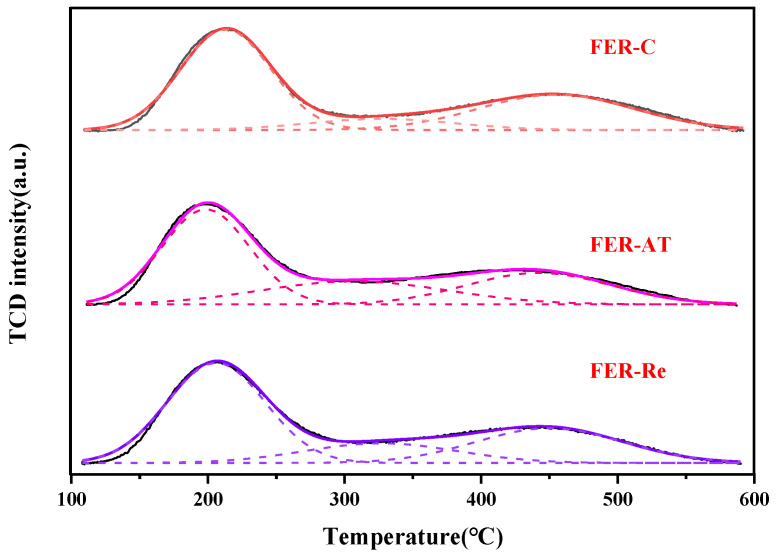
NH_3_-TPD profiles of ferrierite catalysts.

**Figure 5:**
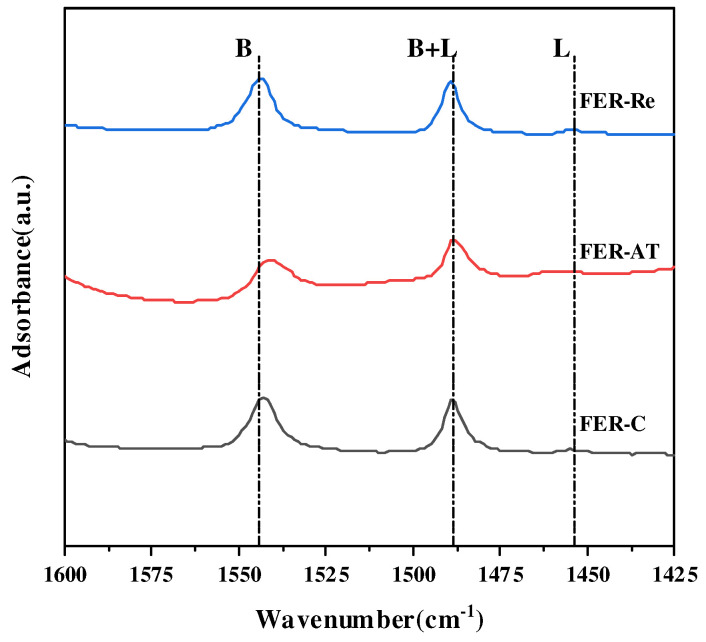
Pyridine FT-IR (Py-IR)spectra at the desorption temperature of 200 °C.

Acid properties of the ferrierite samples were characterized by NH_3_-TPD (Figure 4) and absorbed pyridine infrared spectroscopy (Figure 5), and the results were summarized in Table 2. As shown in Figure 4, all the ferrierite samples showed similar NH_3_-TPD profiles. Three characteristic desorption peaks appeared, corresponding to the weak acid sites at ~200 °C, the medium acid sites at 200–400 °C, and the strong acid sites at 400–600 °C, where the strong acidic sites were well correlated with the number of Brønsted acid sites. The peak positions of the weak and strong acidic sites were not significantly changed on the FER-C and FER-Re. Meanwhile, the peak intensity of FER-AT at same positions declined. Upon alkali treatment, the total acid amount decreased from 1.123 mmol/g of FER-C to 1.040 mmol/g of FER-AT. After recrystallization, the total acid amount in FER-Re increased to 1.221 mmol/g. The changes in the amount of strong acid sites show a similar trend to the total acid amount. A declined amount of strong acid sites from 0.380 mmol/g in FER-C to 0.277 mmol/g occurred in FER-AT, while the recrystallization led to restored strong acid sites of 0.398 mmol/g in FER-Re. Considering that the strong acid sites are widely regarded as the Brønsted acidic sites in zeolite and could be directly reflected by the Si/Al ratio. Thus, the lowest amount of acid sites in FER-AT had the lowest Si/Al ratio which can be ascribed to the pore-blocking effect of the amorphous Si(Al) debris as well as the dealumination caused by the alkali treatment, while more strong acid sites in FER-Re compared with FER-C resulted from its highly crystalline and low-silica nature.

Py-IR measurements were carried out to further investigate the distribution of Brønsted acidic sites in the eight MR and ten MR channels of FER zeolites. As shown in Figure 5, the characteristic bands at 1540 and 1455 cm^−1^ are attributed to the protonated pyridine on Brønsted acid sites and pyridine adsorbed on Lewis acid sites, respectively. The amounts of Brønsted acid sites are calculated by integrating the Py-IR spectra measured at the desorption temperature of 200 °C. As listed in Table 2, FER-AT has less Brønsted acid sites (0.093 mmol/g) compared to FER-C (0.163 mmol/g), while the Brønsted acid sites increased to 0.144 mmol/g in the FER-Re upon recrystallization. Based on the above results, it could be attributed to an increased amount of Brønsted acid sites during the recrystallization step with sodium oleate.

Furthermore, the Brønsted acid sites in eight MR channels on the ferrietes can be quantified by subtracting the amounts of Brønsted acid sites in the ten MR channels analyzed by Py-IR from the total Brønsted acid sites assigned to the strong acidic sites measured by the NH_3_-TPD analysis (Table 2), since the pyridine molecule is too large to be occluded in eight MRs. The amounts of Brønsted acid sites in the eight MR channels were found to be maximized on the FER-Re with its concentration of 0.254 mmol/g and minimized on the FER-AT with that of 0.184 mmol/g, and the amounts of Brønsted acid sites of eight MR on FER-C was 0.217 mmol/g, which are likely to affect catalytic activity.

**Figure 6:**
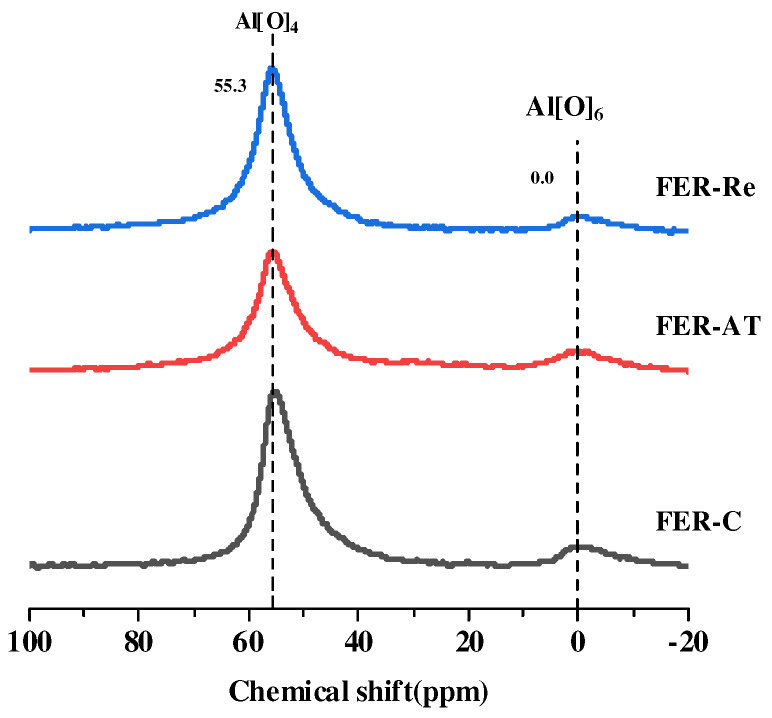
^27^Al MAS NMR spectra of ferrierite catalysts.

Figure 6 shows the ^27^Al MAS NMR spectra of the prepared ferrierite samples. In the ^27^Al MAS NMR spectra, the strong peak at the chemical shift around 55.3 ppm and the weak peak at 0 ppm were referred to as the tetrahedral and octahedral Al species [34], respectively, where the octahedral Al sites are generally related with the extra-framework Al (EFAL) of zeolites. The ratio of EFAL was increased from 7.3% on FER-C to 21.3% on FER-AT due to alkali treatment, and subsequently reduced to 8.3% on FER-Re in the recrystallization process. The trend of the defect rate of Al shows that adding sodium oleate lowers the extra-framework Al species, which get back into the framework during recrystallization by sodium oleate. It was confirmed that the recrystallization utilizing sodium oleate had stonger Brønsted acidity in eight MR.

On the basis of the NH_3_-TPD, Py-IR, and ^27^Al MAS NMR results, the enrichment of Brønsted acid sites in eight MRs after the dissolution–recrystallization process is caused by the preferential reposition of framework Al from ten MR to eight MR with the assistance of sodium oleate.

The research findings suggest that the DME molecule initially interacts with the Brønsted acid sites present in the zeolite, resulting in the formation of methoxy species. Subsequently, this intermediate reacts with CO to produce an acetyl group. It is important to note that the acetyl species located within the eight MR channels can further react with DME, ultimately yielding the desired MA [19,26]. As highlighted by Iglesia et al., the carbonylation reaction of DME is strongly dependent on the quantity of proton sites available within the eight MR channel of the zeolite catalyst. In other words, the number of acidic sites within this specific channel plays a crucial role in facilitating the conversion of DME into MA through the mentioned sequence of reactions [22,35]. The amount of Brønsted acid sites in eight MR of sample FER-Re was larger than that in FER-AT and FER-C. According to reports [27,28,29], stonger Brønsted acidity in eight MR of FER-Re show better catalytic performance in DME carbonylation.

### 2.3. Catalytic Activity Test

**Figure 7:**
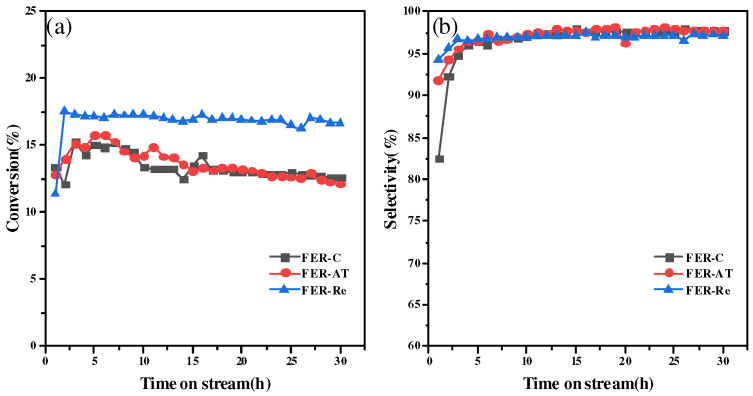
(**a**) The conversion of DME and (**b**) selectivity to MA during 30 h time-on-stream test on ferrierite catalysts, 200 °C, 2.0 MPa.

The catalytic performance including conversion of DME and selectivity to MA with time on stream for 30 h reaction are displayed in Figure 7. As shown in Figure 7a, the parent ferrierite FER-C presents the lowest DME conversion of 14%, which is slightly lower than the activity over the alkali-treated sample FER-AT. Recrystallized FER-Re presents the highest conversion of DME (up to 17%) among three catalysts. The conversion of DME over FER-Re catalyst was stable during the 30 h time-on-stream test, while the DME conversion of FER-C and FER-AT increased for the first six hours and then went down for the next 24 h. After 30 h reaction, the 3% conversion gap between FER-Re and others was still maintained.

Furthermore, it also presents the excellent selectivity to MA, as well as FER-C and FER-AT. The higher conversion of DME to MA during the 30 h time-on-stream test were observed on the FER-Re compared to those of the FER-C and FER-AT. Furthermore, supporting experimental evidence such as NH3-TPD profiles, SEM, and TEM photos were combined with the observed catalytic results. This analysis suggested that FER-Re possessed a larger number of Brønsted acid sites within the eight MR channels due to its higher crystallinity and more regular shape, which is consistent with experimental results reported in the literature. Combined with the experimental results of NH_3_-TPD profiles, SEM, and TEM photos, the excellent catalytic performance of the FER-Re was speculated to be due to the fact that FER-Re has a larger number of Brønsted acid sites in the eight MR channels due to its higher crystallinity and regular shape, which is consistent with experimental results reported in the literature [3,21,28,29,36]. According to the spectra of ^27^Al MAS NMR, it was presumed that sodium oleate may have affected the co-ordination of Al in the post-treatment, and thus led to acid sites distribution. Overall, based on the experimental results and characterizations, it can be speculated that the superior catalytic performance of FER-Re is attributed to its increased number of Brønsted acid sites resulting from higher crystallinity and a more regular shape.

## 3. Materials and Methods

### 3.1. Catalyst Preparation

Commercially available FER zeolites (denoted as FER-C) were provided by Shanghai Petrochemical Research Institute (SPRI). In the post-treatment process, ferrierites were treated with 0.3 M NaOH (96.0 wt%, China National Medicine Group, Shanghai Chemical Reagent Co., Shanghai, China) solution at 80 °C for 6 h with solution/zeolite ratio of 10:1. After that, the suspension was cooled down and washed by deionized water thoroughly before being dried overnight. The obtained samples were denoted as FER-AT. Recrystallized FER zeolites (denoted as FER-Re) were prepared with a tow-step produce including etching the zeolite in 0.3 M NaOH solution followed by the hydrothermal recrystallization in the presence of 0.1 M sodium oleate (China National Medicine Group, Shanghai Chemical Reagent Co.) at 170 °C for 12 h. Products were recovered by filtration, washed thoroughly with distilled water, and dried overnight. Then, all the FER samples were calcined at 550 °C for 4 h. To convert the FER zeolites into H-form, the samples were exchanged with 10 wt% CH_3_COONH_4_ solution at 80 °C for 2 h three times and calcined in a dry air flow at 550 °C for 4 h.

### 3.2. Catalyst Characterization

The X-ray diffraction (XRD) patterns were obtained using a D8 Advance SS X-ray diffractometer with Cu Kα radiation at an operational voltage of 40 kV and current of 40 mA. Scanning electron microscopy (SEM) images were captured using a Hitachi S-4800 microscope, operating at an accelerated voltage of 3.0 kV. The transmission electron microscopy (TEM) images were acquired on a JEOL JEM-2100F electron microscope, operating at 200 kV. ICP-AES measurements were conducted utilizing a Thermo IRIS Intrepid II XSP atomic emission spectrometer. The specific surface area was determined by employing the BET method. To perform ammonia temperature-programmed desorption (NH3-TPD), an AMI-3300 apparatus from Altamira Instruments, equipped with a thermal conductivity detector (TCD), was utilized. Infrared (IR) spectra of pyridine adsorption were recorded using a Nicolet NEXUS 670 FT-IR spectrometer. The spectra were collected at 200 °C after subjecting the samples to vacuum treatment at 400 °C. Solid-state magic angle spinning nuclear magnetic resonance (MAS NMR) spectra of 27Al were obtained on a JNM-ECZ500R/S1 spectrometer manufactured by JEOL, employing a one-pulse condition.

### 3.3. Catalytic Experiments

The catalyst performance in the DME carbonylation reaction was evaluated using a fixed bed reactor. Before the reaction, the catalyst underwent a pretreatment step where it was exposed to a nitrogen flow at 400 °C for 2 h to remove any water present.

The reaction was performed at 200 °C and 2.0 MPa, with a DME weight hourly space velocity (WHSV) of 0.1 h^−1^. The DME and CO mixed feed, with a molar ratio of nCO/nDME = 10/1, occurred when the temperature inside the reactor decreased to 200 °C, and a gas mass flow controller (MFC) was used to control the flow rate of the gas mixture

## 4. Conclusions

In conclusion, we have developed an innovative method for recrystallizing ferrierites using a special chelating ligand, such as sodium oleate. This approach successfully regulated the distribution of acid sites by influencing ions other than alkali metals. The resulting recrystallized ferrierite, FER-Re, prepared in the presence of sodium oleate, exhibited excellent catalytic performance in the DME carbonylation reaction. Characterization results from SEM, TEM images, Py-IR, NH3-TPD, and ^27^Al MAS NMR indicated that sodium oleate played a crucial role during hydrothermal alkali treatment in protecting the ferrierite zeolite from alkali damage. It contributed to a higher crystallinity and a more regular shape of the catalytically effective FER-Re. Moreover, sodium oleate chelates some extra-framework Al species that are attacked by alkali in ferrierite, increasing the amount of Brønsted acid sites as well as catalytically active sites in the eight MR channels of ferrierites, which directly leads to a higher efficiency of MA carbonylation. This research work is of pioneering significance to the synthesis of zeolites and provides a novel way of postprocessing, which is expected to be extended to the preparation of other types of zeolites.

## Figures and Tables

**Table 1:** Characteristics of ferrierites after ion exchange.

Samples	Relative Crystallinity ^a^	Si/Al ^b^	N_2_ Sorption				
			Surface Area (m^2^/g)			Pore Volume (cm^3^/g)	
			S_BET_	S_mic_	S_ext_	V_micro_	V_meso_
FER-C	1.0	20.7	342.35	330.44	41.91	0.130	0.136
FER-AT	0.80	18.9	289.24	230.14	59.10	0.104	0.178
FER-Re	0.96	20.3	330.09	279.08	51.01	0.124	0.160

^a^ Estimated from the intensity of the diffraction peak at 2θ =9.44°, using the parent ferrierite as a reference. ^b^ Determined by ICP.

**Table 2:** Surface acidic properties.

Samples	^27^Al NMR ^a^	NH_3_-TPD		Py-IR ^b^		B Sites in 8 MR ^c^
	EFAL	Total Acid /(mmol/g)	Weak/Medium/Strong (mmol/g)	Brønsted (mmol/g)	Lewis (mmol/g)	
FER-C	7.3	1.123	0.630/0.113/0.380	0.163	0.0056	0.217
FER-AT	21.3	1.040	0.502/0.261/0.277	0.093	0.0087	0.184
FER-Re	8.3	1.221	0.666/0.157/0.398	0.144	0.0090	0.254

^a^ Defect ratio on the all FERs was calculated from ^27^Al MAS NMR peak after its separate deconvolution of tetrahedral peak at ~55.3 ppm and octahedral peak at ~0 ppm, which was defined as [(Area of octahedral Al peak area)/(Sum of octahedral and tetrahedral Al peak area)] × 100%. ^b^ Amounts of the Brønsted (B) and Lewis (L) acid sites were measured by pyridine FT-IR (Py-IR) at the adsorption temperature of 200 °C, and the number of acidic sites were calculated using the well-known formula of B sites = 1.88 A_B_R^2^/W, L sites = 1.42 A_L_R^2^/W [33], where A_B_ and A_L_ stand for the integrated absorbance peak area of Brønsted and Lewis acid sites located at about 1540 and 1455 cm^−1^, and R represents the radius of catalyst pellet with catalyst weight (W) of the all FERs. ^c^ Overall number of Brønsted acid sites (strong acid sites analyzed by NH_3_-TPD)—number of Brønsted acid sites in the 10 MR channels characterized by Py-IR analysis.

## Data Availability

Not applicable.

## Sample Availability

Samples of the compounds are available from the authors.
